# Associations between genetic variants in sphingolipid metabolism pathway genes and hepatitis B virus-related hepatocellular carcinoma survival

**DOI:** 10.3389/fonc.2023.1252158

**Published:** 2024-01-08

**Authors:** Binbin Jiang, Moqin Qiu, Liming Qin, Jingmei Tang, Shicheng Zhan, Qiuling Lin, Junjie Wei, Yingchun Liu, Zihan Zhou, Xiumei Liang, Ji Cao, Jiawei Lian, Yuejiao Mai, Yanji Jiang, Hongping Yu

**Affiliations:** ^1^ Department of Experimental Research, Guangxi Medical University Cancer Hospital, Nanning, China; ^2^ Department of Respiratory Oncology, Guangxi Medical University Cancer Hospital, Nanning, China; ^3^ Department of Epidemiology and Health Statistics, School of Public Health, Guangxi Medical University, Nanning, China; ^4^ Department of Clinical Research, Guangxi Medical University Cancer Hospital, Nanning, China; ^5^ Department of Cancer Prevention and Control, Guangxi Medical University Cancer Hospital, Nanning, China; ^6^ Department of Disease Process Management, Guangxi Medical University Cancer Hospital, Nanning, China; ^7^ Department of Scientific Research Dept, Guangxi Medical University Cancer Hospital, Nanning, China; ^8^ Key Laboratory of Early Prevention and Treatment for Regional High Frequency Tumor, Guangxi Medical University, Ministry of Education, Nanning, China; ^9^ Guangxi Health Commission, Key Cultivated Laboratory of Cancer Molecular Medicine, Guangxi Medical University Cancer Hospital, Nanning, China

**Keywords:** hepatocellular carcinoma, sphingolipid metabolism, GBA2, single nucleotide polymorphism, overall survival

## Abstract

**Background:**

Although the sphingolipid metabolism pathway is known to play a significant role in tumor progression, there have been few studies on how genetic variants in the sphingolipid metabolism pathway genes affect the survival of patients with hepatitis B virus (HBV)-related hepatocellular carcinoma (HCC).

**Methods:**

We utilized available genotyping data to conduct multivariate Cox proportional hazards regression model analysis, examining the associations of 12,188 single nucleotide polymorphisms (SNPs) in 86 sphingolipid metabolism pathway genes on the survival of 866 HBV-HCC patients, and the model was also used in additive interaction analysis. We used bioinformatics functional prediction and expression quantitative trait locus (eQTL) analysis to explore the potential functions of SNPs and to evaluate the association of SNPs with the corresponding mRNA expression, respectively. We also used the online database TIMER2.0 (http://timer.comp-genomics.org/) to analyze the relationship between the corresponding mRNA expression levels and immune cell infiltration.

**Results:**

Our study found that *GBA2* rs1570247 G>A was significantly associated with elevated survival of HBV-HCC patients [(hazards ratio (HR*)*=0.74, 95% confidence interval (CI*)*=0.64-0.86, *P*<0.001)]. And on an additive scale, a synergistic effect was observed between the GG genotype of rs1570247 and advanced BCLC stage. Among HBV-HCC patients with advanced BCLC stage, those carrying the GBA2 rs1570247 GG genotype exhibited a significantly elevated risk of mortality (HR=3.32, 95%CI=2.45-4.50). Further functional prediction and eQTL analysis revealed that rs1570247 were located in the 5’ untranslated region of the *GBA2*, the A allele of SNP rs1570247 was associated with higher mRNA expression levels of *GBA2* in normal liver tissues (*P*=0.009). Moreover, we observed a positive correlation between *GBA2* mRNA expression and the infiltration level of B lymphocytes cell (*R*=0.331, *P*<0.001), while a negative correlation was noted between *GBA2* mRNA expression and the infiltration level of macrophage M2 in HCC (*R*=-0.383, *P*<0.001).

**Conclusion:**

Our findings suggest that GBA2 rs1570247 G>A in sphingolipid metabolism pathway may be a key factor for survival of HBV-HCC patients by regulating the expression of corresponding genes and affecting the infiltration level of immune cells.

## Introduction

Hepatocellular carcinoma (HCC) is one of the most common cancers worldwide and represents a major challenge to global healthcare. In 2020, nearly 906,000 people were diagnosed with liver cancer globally, mostly HCC (1). Unfortunately, HCC is also the third highest cause of cancer-related deaths, with a poor 5-year survival rate of around 15% (2). It’s worth noting that most patients with HCC have a background of hepatitis B virus infection (HBV) in Asia, especially in in Guangxi Province, Sorthern China, the infection rate was as high as 87.7% (3).

Despite surgical procedure for operable HCC patients can lead to a better 5-year survival of over 70%, there is still a large part of patients suffering from poor survival because of high recurrence or metastasis rates (4). In patients with operable HCC, their genetic backgrounds are also likely influence their survival (5, 6). Although several survival-predicting genetic variants have identified in genome-wide association studies (7–9), these variants do not provide clear biological relevance. Recent pathway analyses for identifying survival-predicting genetic variants appear to be effective and found several additional variants that are potentially functional (10–12). Therefore, identifying efficient prognostic biomarkers is still urgent for identifying patients with high-death risk and for strategy-decision making of personalized treatment in operable HBV-HCC patients.

Sphingolipids are one of the main categories of lipids in eukaryotic organisms, participating in the composition of the plasma membrane and serving as regulators of cell-cell interactions and cell recognition (13). Bioactive sphingolipids are now recognized as important regulators of cancer cell biology. For example, sphingosine-1-phosphate can promote tumor cell growth through Stat3 and Akt signaling pathways, upregulate Bcl-2/Bcl-xL, and inhibit p53-mediated apoptosis (14–18). Ceramides, on the other hand, promote tumor progression through chronic inflammation and endoplasmic reticulum stress (19, 20). Additionally, sphingolipids are closely associated with tumor immune responses. Sphingosine-1-phosphate and lysophosphatidic acid can modulate the anti-tumor activity of infiltrating lymphocytes, and the acidic tumor microenvironment can activate acid sphingomyelinase and induce matrix metalloproteinase-9, thereby promoting tumor metastasis and immune evasion (21–24). Studies have also reported that sphingolipids in hepatocellular carcinoma (HCC) can influence patient prognosis by mediating the immune microenvironment(25). Specifically, abnormal ceramides produced by liver cells infected with HBV PreS variants can activate NLRP3 inflammasome in hepatic macrophages, promoting the progression of hepatocellular carcinoma (26). Furthermore, elevated plasma ceramide levels in pancreatic cancer can predict the response to radiotherapy in metastatic tumors; similarly, Ceramides6 has emerged as a strong prognostic factor for survival in human colon cancer patients (27, 28). Therefore, potential genetic variations in sphingolipid metabolic pathway genes may serve as promising predictive biomarkers for HCC patient survival.

In the presen study, we hypothesize that genetic variants in sphingolipid metabolism pathway genes are associated with survival of HBV-HCC patients. We tested this hypothesis by using genotyping data from HBV-HCC patients and focusing on SNPs that may alter their gene function or expression levels and are therefore likely to have biological and functional implications.

## Materials and methods

### Study populations

In this study, a total of 866 HBV-HCC patients were enrolled. These patients were HBsAg seropositive HCC patients with histologically confirmed operable HCC and underwent hepatectomy at the Guangxi Medical University Cancer Hospital between July 2007 and December 2017. The eligibility criteria and clinical characteristics of all patients have been previously described(6). The investigators collected the general information [i.e age, sex, smoking status, drinking status, serum AFP level, cirrhosis, embolus, and the Barcelona Clinic Liver Cancer (BCLC) stage] and follow-up information of patients by reviewing medical records and telephone follow-up. All patients were followed up every three months within the first two years after surgery and every six months in the next year through telephone calls. Overall survival (OS) was defined as the time from surgery to death or to the date of the last follow-up (March 2020). Patients who were lost to follow-up or survived at the last follow-up were censored. Because this was a single institution study, we used an internal study validation design to ensure the accuracy and reliability of the results, the 866 cases were randomly divided into a discovery group and a replication group at a ratio of 1:1. The present study was approved by Guangxi Medical University Cancer Hospital Institutional Review Committee (LW2023109).

### Genotyping, gene and SNP selection

The method used for genotyping SNPs, the quality control of raw data and imputation have been described elsewhere (5). In brief, the whole genomic DNA of each HCC patient was extracted by a blood DNA extraction kit (Concert). Genotyping was performed using Illumina Infinium Global Screening Assay (Shanghai, China). Based on the 1000 Genomes Project (Phase 3 v5) reference population information, Minimac3 was used for imputation (https://imputationserver.sph.umich.edu/index.htm)forthe untyped SNPs.

The sphingolipid metabolism pathway-related genes were gathered from the Molecular Signatures Database (MSigDB) (http://www.broadinstitute.org/gsea/msigdb/index.jsp) using the keyword “sphingolipid”. After eliminating 37 duplicates and six genes located in the X chromosome, a total of 86 genes were selected as candidates (**Supplementary Table S1**).

After quality control and imputation, all SNPs in these genes and their ±2 kb flanking regions were extracted by Plink 1.09 (http://pngu.mgh.harvard.edu/purcell/plink/)(29) based on the following criteria: minor allele frequency (MAF) ≥ 0.05, the genotyping success rate was ≥ 95%, and Hardy-Weinberg equilibrium (HWE) was ≥ 1.0 × 10^-6^. As a result, a total of 12,188 SNPs were obtained from the discovery dataset for further analysis.

### Functional annotation and eQTL analysis

Functional prediction of the identified SNPs was carried out using three online tools: SNPinfo (https://snpinfo.niehs.nih.gov/snpinfo/snpfuNc.htm), RegulomeDB (http://www.regulomedb.org/), and VannoPortal (http://www.mulinlab.org/vportal). The linkage disequilibrium (LD) of the selected SNPs was analyzed and LD plots were generated using Haploview software.

To assess the relationships between different genotypes of SNPs and the mRNA expression levels of corresponding genes, an expression quantitative trait locus (eQTL) analysis was performed using the GTEx database (https://www.gtexportal.org/). The relationships between the expression levels of target genes and the infiltration levels of multiple immune cells in the tumor microenvironment were analyzed using the tumor immune cell infiltration database TIMER2.0 (http://timer.comp-genomics.org/). Additionally, the mRNA expression of target genes in HCC tissues and normal tissues and their association with survival of HCC patients were analyzed through the Kaplan-Meier Plotter (https://kmplot.com/) database.

### Statistical analysis

To evaluate the association between SNPs and survival in 866 HBV-HCC patients after surgery, a multivariate Cox proportional hazards regression was performed using the GenABEL package in R 3.1.3. Bayesian false discovery probability (BFDP) was used to correct for the incidence of false positive errors (30). The effect of different SNP genotypes on postoperative OS in patients with HBV-HCC was analyzed using the survival package in R 4.0.3, and Kaplan-Meier curves were generated.

In order to test the reliability of the association analysis results, we used the bootstrap method to calculate the hazard ratio (HR) of each time by 1,000 repeated sampling. The Shapiro-Wilk test was utilized to assess the fitting degree and 95% confidence interval (CI) of the normal distribution of HR values, thereby evaluating the reliability of the study results.

## Result

### Associations of SNPs in the sphingolipid metabolism pathway with survival

**Figure 1:**
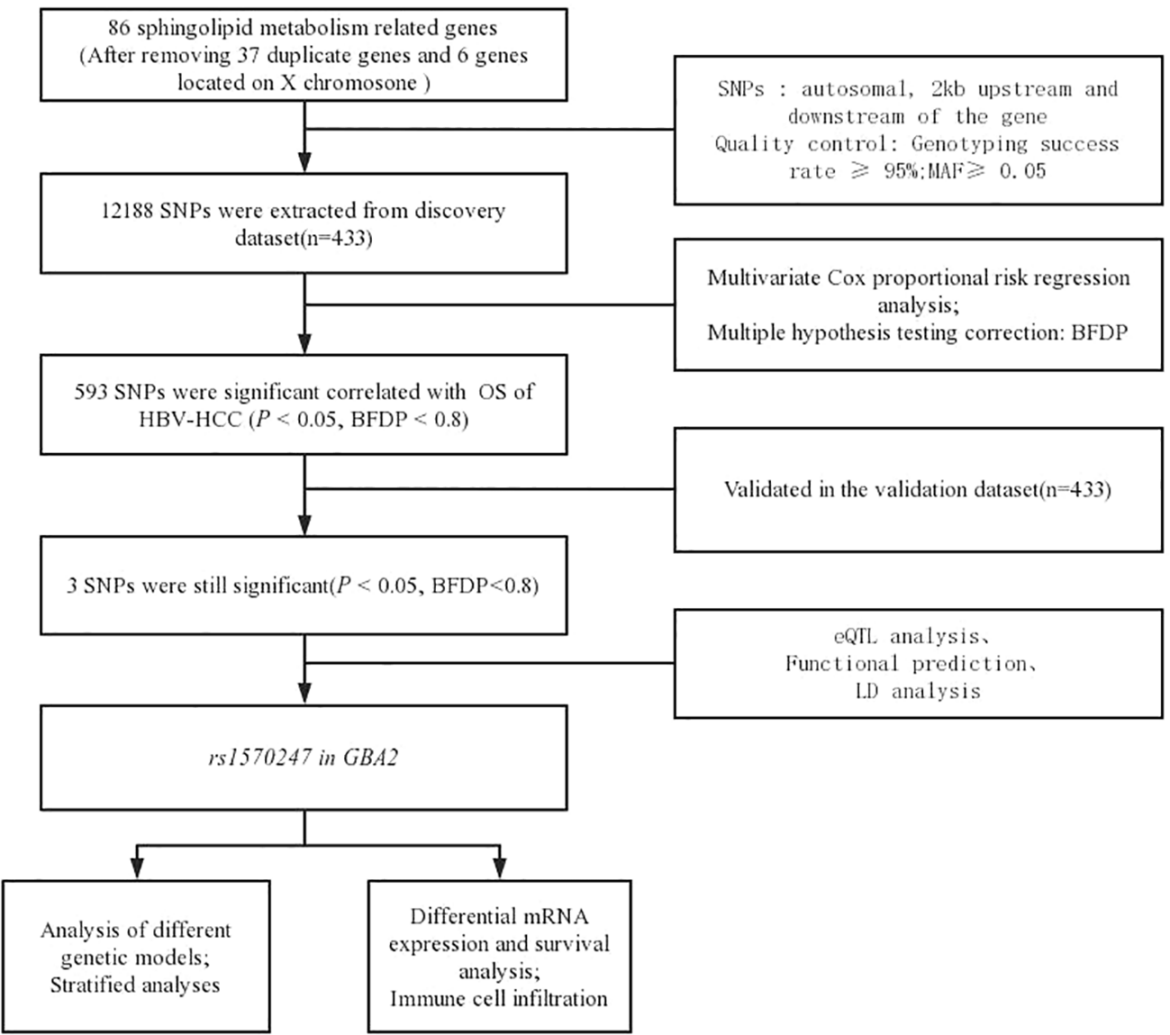
Flow chart of the analysis process.

**Figure 1** illustrates the flowchart of the study design. The discovery dataset consisted of 12,188 SNPs in 86 genes related to sphingolipid metabolism pathway, among which 593 SNPs were found to be associated with HBV-HCC OS (*P*<0.05, BFDP<0.8). After verification in the replication dataset, 3 SNPs (rs1570246 G>T, rs1570247 G>A, rs3750434 G>A) located on glucosylceramidase beta 2 (*GBA2*) remained statistically significant (*P*<0.05, BFDP<0.8) (**Table 1**).

**Table 1:** Associations of 3 validated significant SNPs with HBV-HCC OS in discovery, validation and combined dataset.

SNPs	Gene	Allele	MAF	Discovery dataset(n=433)			Replication dataset(n=433)		Combined dataset(n=866)		
				HR(95%CI)	*P* ^a^	BFDP	HR(95%CI)	*P* ^a^	HR(95%CI)	*P* ^a^	BFDP
rs1570247	*GBA2*	G>A	0.435	0.67(0.54-0.83)	<0.001	0.421	0.80(0.65-0.99)	0.037	0.74(0.64-0.86)	<0.001	0.228
rs1570246	*GBA2*	G>T	0.449	0.76(0.54-0.82)	<0.001	0.252	0.81(0.66-1.00)	0.047	0.74(0.64-0.86)	<0.001	0.228
rs3750434	*GBA2*	G>A	0.445	0.71(0.57-0.87)	0.001	0.698	0.79(0.65-0.96)	0.020	0.76(0.65-0.87)	<0.001	0.201

a Adjusted for age, sex, smoking, drinking, AFP, cirrhosis, embolus, BCLC stage in Cox regression models.

SNP, single nucleotide polymorphisms; MAF, minor allele frequency; HR, hazards ratio; CI, confidence interval; BFDP, Bayesian false discovery probability.

### LD analysis and functional annotation

**Figure 2:**
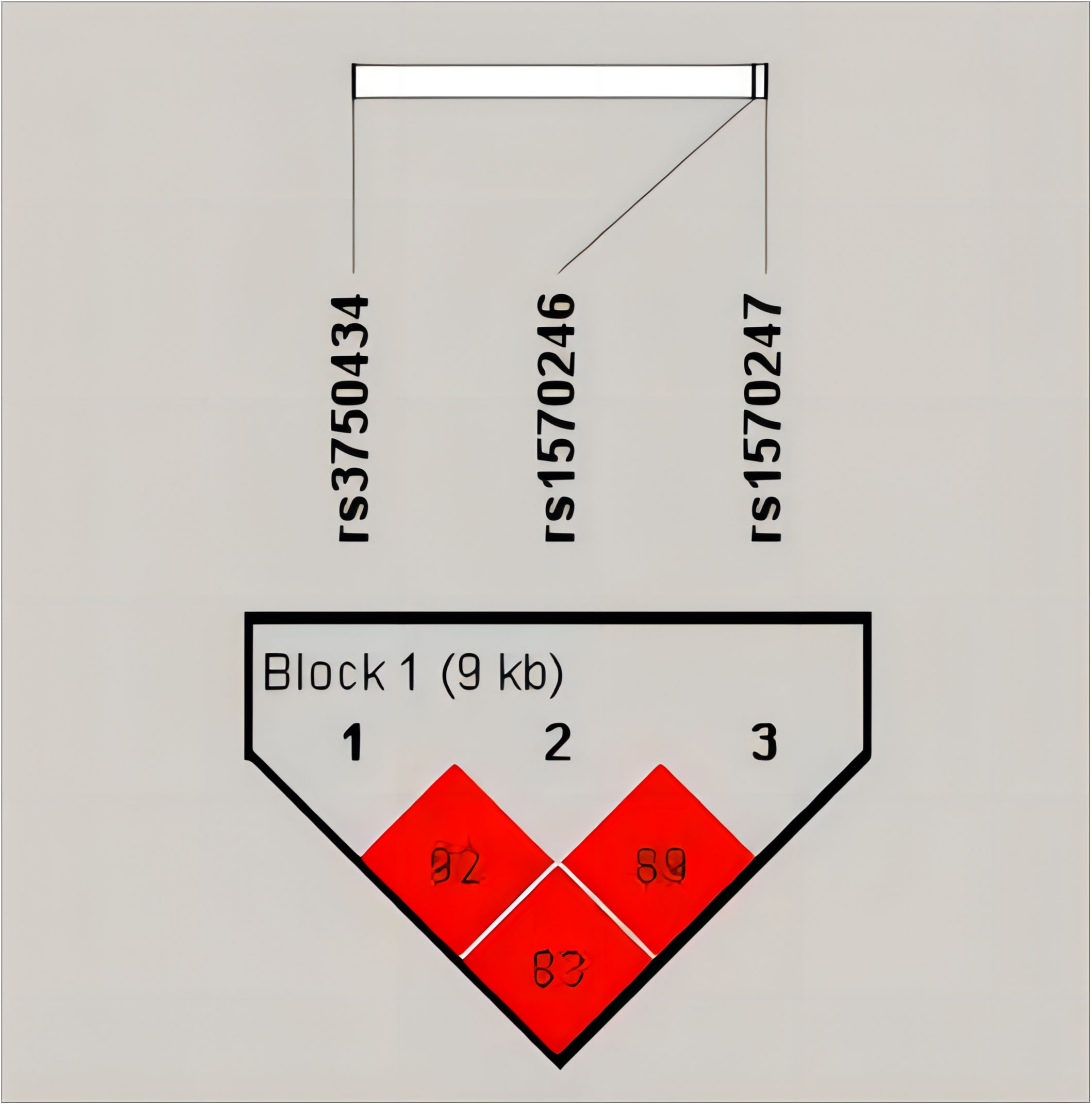
Linkage disequilibrium analysis.

The results of LD analysis revealed high linkage (*r^2^* >0.8) between these three SNPs in *GBA2* gene (**Figure 2**). To select a tagSNP with potential functions, we performed functional annotation for these three SNPs using RegulomeDB, SNPinfo, and VannoPortal. As shown in **Table 2**, rs1570246 and rs1570247 were located in the 5’ untranslated region (UTR) of the *GBA2* gene, while rs3750434 was located in the intronic region. Importantly, the rs1570247 with the highest potential functional score and the highest context-dependent priority was located in an active region of chromatin with many histone modifications, indicating a high probability of potential function. Therefore, we selected rs1570247 for subsequent analysis.

**Table 2:** Functional prediction of 3 significant SNPs associated with HCC OS.

Variant	rsID	Gene	Location	RegDB ^a^	SNPinfo ^b^	VannoPortal ^c^		
						REG Score	Mark	Comb Rank in LD
9:35749014 G|A	rs1570247	*GBA2*	5’UTR	2a	TFBS	0.985	H3K79me2,H3K4me3,H3K27ac,DNase	1.5
9:35748806 G|T	rs1570246	*GBA2*	5’UTR	4	TFBS	0.978	H3K4me2,H3K79me2,H3K4me3,H3K27ac,DNase	2.3
9:35739876 G|A	rs3750434	*GBA2*	intronic	5	–	0.894	H3K36me3	5.6

a RegulomeDB 2.0: https://www.regulomedb.org/.

b SNPinfo: https://manticore.niehs.nih.gov/.

c VannoPotal: http://www.mulinlab.org/.

5’UTR, 5’Untlanslated Region; TFBS, Transcription factors binding site.

### Genetic models

To assess the impact of SNPs on the prognosis of HBV-HCC patients, we applied multivariable Cox regression to evaluate the effect of rs1570247 on the risk of death in the combined dataset. The results revealed a dose-response relationship between the A allele amount of rs1570247 and decreased risk of death in patients (**Table 3**).

**Table 3:** Associations between rs1570247 and postoperative OS of HBV-HCC patients in different genetic models of all datasets.

Genetype	Discovery dataset (n=433)				Replication dataset (n=433)				Combined dataset (n=866)			
	Cases	Deaths (%)	HR(95%CI)	*P* ^a^	Cases	Deaths (%)	HR(95%CI)	*P* ^a^	Cases	Deaths (%)	HR(95%CI)	*P* ^a^
GG	129	72(55.8)	1.00		148	88(59.5)	1.00		277	160(57.8)	1.00	
GA	213	89(41.8)	0.50(0.36-0.70)	<0.001	218	100(45.9)	0.66(0.49-0.88)	0.004	431	189(43.9)	0.59(0.48-0.73)	<0.001
AA	91	39(42.9)	0.49(0.33-0.73)	<0.001	67	31(46.3)	0.75(0.50-1.14)	0.182	158	70(44.3)	0.62(0.47-0.82)	<0.001
*P*_trend_				<0.001				0.037				<0.001
GG	129	72(55.8)	1.00		148	88(59.5)	1.00		277	160(57.8)	1.00	
GA+AA	303	128(42.2)	0.50(0.37-0.68)	<0.001	285	131(50.0)	0.68(0.51-0.89)	0.005	589	259(44.0)	0.60(0.49-0.73)	<0.001

a Adjusted for age, sex, smoking, drinking, AFP, cirrhosis, embolus, BCLC stage in Cox regression models.

HR, hazards ratio; CI, confidence interval.

**Figure 3:**
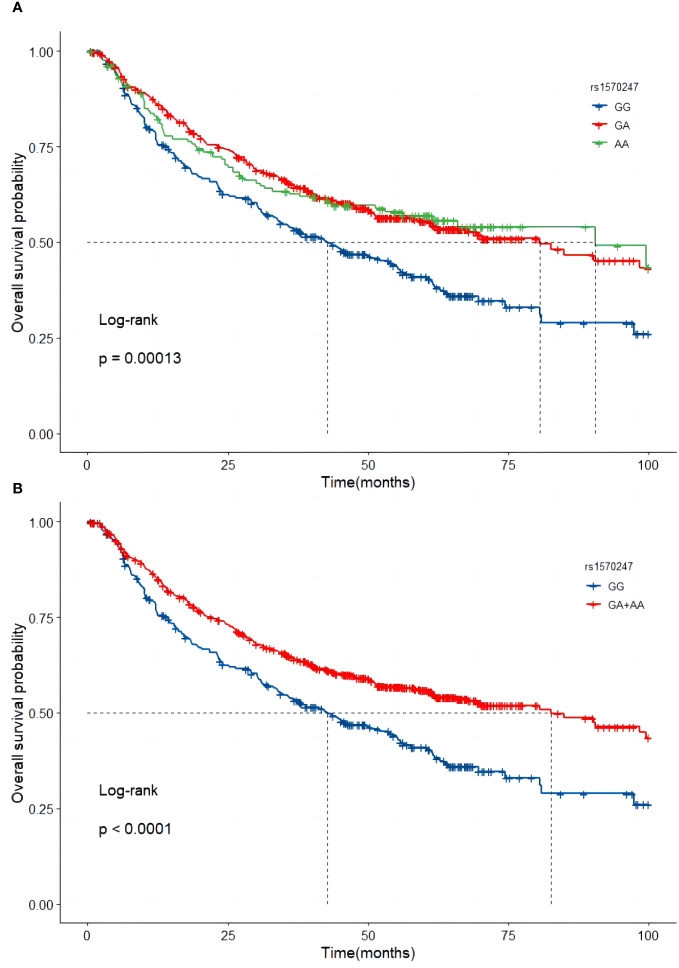
Kaplan-Meier curves of *GBA2* rs1570247 in combined dataset. **(A)** Additive model; **(B)** Dominant model.

In the combined dataset, the mortality rates for different genotypes of *GBA2* rs1570247 were 57.8% for GG, 43.9% for GA, and 44.3% for AA. Patients with the rs1570247 GA or AA genotype had better survival rates than those with the GG genotype (*HR*=0.60, 95%*CI*=0.49-0.73, *P*<0.001). Kaplan-Meier survival curves were generated to illustrate the associations between rs1570247 and OS in patients with HBV-HCC (**Figure 3**).

**Figure 4:**
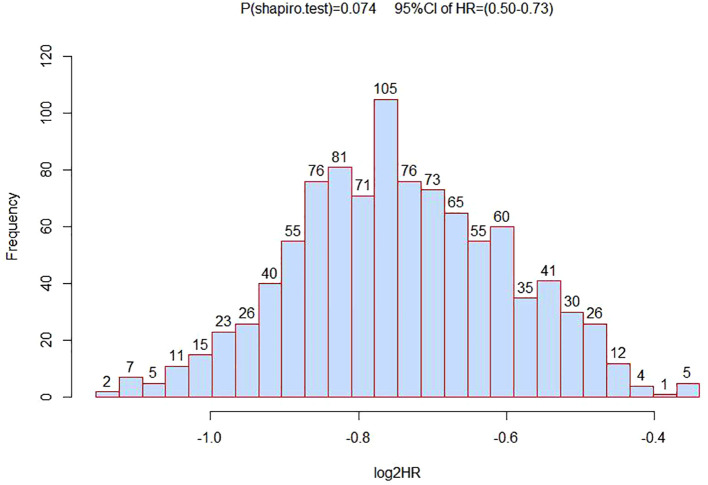
The distribution histogram of 1000 HR values after grouped by bootstrapping for 1000 times.

To verify the association analysis results, we conducted 1,000 repeated samplings using the Bootstrap method and performed multivariate Cox regression analysis to calculate HR and 95% CI. The distribution of HR in verification was consistent with a normal distribution (*P*<0.05). And the HR value for rs1570247 in the survival analysis of HCC patients was within the 95% confidence interval (**Figure 4**). These observations suggested the overall reliability of association analysis.

### Stratified analysis

**Figure 5:**
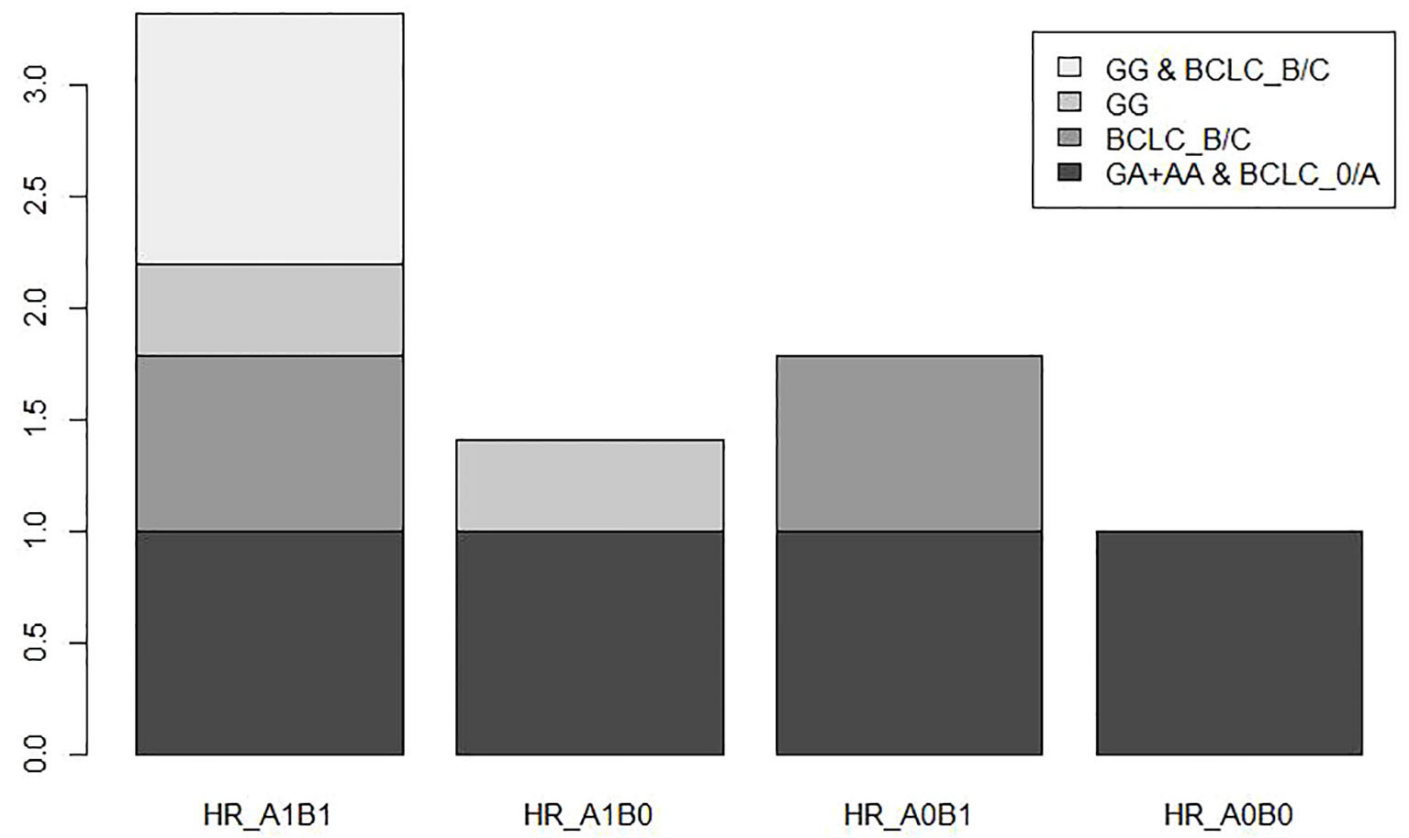
Additive interaction between BCLC stage and rs1570247.

To further investigate the association between different genotypes and OS in the 866 cases, we performed a stratified analysis using covariates such as age, sex, smoking, drinking, AFP, cirrhosis, embolus, and BCLC stage as stratification factors. Obtained results revealed that the GA and AA genotypes of rs1570247 were associated with a significantly reduced risk of postoperative death in multiple subgroups, except for female patients (**Table 4**). Then, we selected 4 clinical variables that were statistically significant for HBV-HCC survival in our dataset to analyze the interaction with rs1570247 on an additive scale. Our results showed a synergistic effect between rs1570247 GG genotype and HBV-HCC patients with advanced BCLC stage on an additive scale (**Table 5**). Specifically, HBV-HCC patients with GG genotype in advanced BCLC stage have a higher risk of death(HR=3.32, 95%CI=2.45-4.50). The visualization results were shown in **Figure 5**.

**Table 4:** Stratification analysis of rs1570247 genotypes with postoperative OS of HBV-HCC.

Variables	GG		GA+AA		Multivariate analysis^b^	
	Cases	Deaths (%)	Cases	Deaths (%)	HR(95%CI)	*P* ^a^
Age						
≤47	130	83(63.8)	304	150(49.3)	0.57(0.43-0.75)	<0.001
>47	147	77(52.4)	285	109(38.2)	0.65(0.48-0.87)	0.004
Sex						
Female	28	12(42.9)	78	30(38.5)	0.67(0.33-1.38)	0.278
Male	249	148(59.4)	511	229(44.8)	0.59(0.48-0.73)	<0.001
Smoking						
Never	168	95(56.5)	377	173(45.9)	0.66(0.51-0.86)	0.002
Ever	109	65(59.6)	212	86(40.6)	0.51(0.37-0.71)	<0.001
Drinking						
Never	192	104(54.2)	422	188(44.5)	0.65(0.51-0.83)	<0.001
Ever	85	56(65.9)	167	71(42.5)	0.46(0.32-0.67)	<0.001
AFP						
≤400	172	93(54.1)	350	139(39.7)	0.64(0.49-0.83)	<0.001
>400	105	67(63.8)	239	120(50.2)	0.57(0.42-0.78)	<0.001
Cirrhosis						
No	122	73(59.8)	269	112(41.6)	0.54(0.40-0.74)	<0.001
Yes	156	88(56.4)	320	147(45.9)	0.63(0.48-0.82)	<0.001
Embolus						
No	205	101(49.3)	431	159(36.9)	0.63(0.49-0.81)	<0.001
Yes	72	59(81.9)	158	100(63.3)	0.54(0.38-0.76)	<0.001
BCLC Stage						
0/A	132	53(40.2)	295	93(31.5)	0.69(0.49-0.97)	0.030
B/C	145	107(73.8)	294	166(56.5)	0.55(0.43-0.72)	<0.001

a Adjusted for age, sex, smoking, drinking, AFP, cirrhosis, embolus, BCLC stage in Cox regression models.

b Multiplicative interaction between rs1570247 and clinical variables.

HR, hazards ratio; CI, confidence interval.

**Table 5:** Additive interaction between rs1570247 and clinical variables.

Variables	Genetype		HR(95%CI)	AP(95%CI)	RERI(95%CI)			S(95%CI)
Age								
>47	GA+AA	1.00		0.19(-0.07-0.46)		0.42(-0.20-1.05)	1.56(0.74-3.28)	
≤47	GA+AA	1.21(0.94-1.56)						
>47	GG	1.54(1.15-2.07)						
≤47	GG	2.17(1.63-2.90)						
AFP								
≤400	GA+AA	1.00		0.25(-0.01-0.51)		0.58(-0.10-1.26)	1.78(0.85-3.76)	
>400	GA+AA	1.22(0.95-1.58)						
≤400	GG	1.52(1.17-1.98)						
>400	GG	2.32(1.73-3.12)						
Embolus								
No	GA+AA	1.00		0.26(0.00-0.51)		0.79(-0.15-1.73)	1.61(0.93-2.79)	
Yes	GA+AA	1.70(1.28-2.26)						
No	GG	1.60(1.24-2.05)						
Yes	GG	3.08(2.22-4.29)						
BCLC Stage								
0/A	GA+AA	1.00		0.34(0.13-0.54)		1.12(0.30-1.93)	1.93(1.12-3.33)	
B/C	GA+AA	1.79(1.34-2.39)						
0/A	GG	1.41(1.01-1.98)						
B/C	GG	3.32(2.45-4.50)						

HR, hazards ratio; CI, confidence interval; AP,attributable proportion due to interaction; RERI, relative excess risk due to interaction; S, the synergy index.

### eQTL analysis, differential mRNA expression analysis and survival analysis

**Figure 6:**
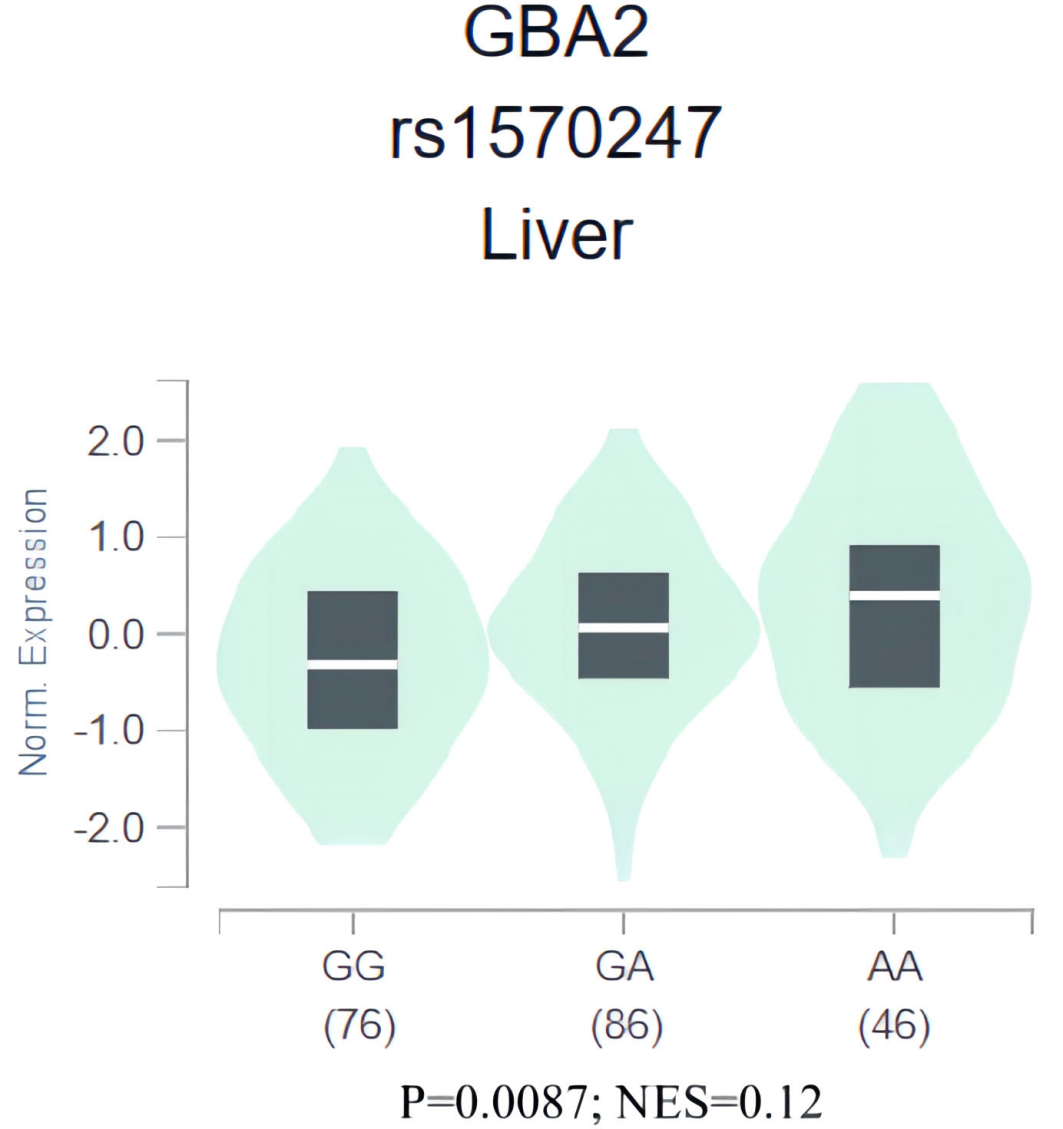
eQTL analysis of rs1570247 and *GBA2* mRNA expression levels in liver tissues from GTEx database.

To gain further insight into the role of rs1570247 in HCC, we conducted an eQTL analysis in the GTEx database to investigate the association between rs1570247 genotype and *GBA2* mRNA expression levels. Our results showed that the rs1570247 AA genotype was significantly associated with increased *GBA2* mRNA expression levels in normal liver tissues (*P*=0.0087, NES=0.12) (**Figure 6**).

**Figure 7:**
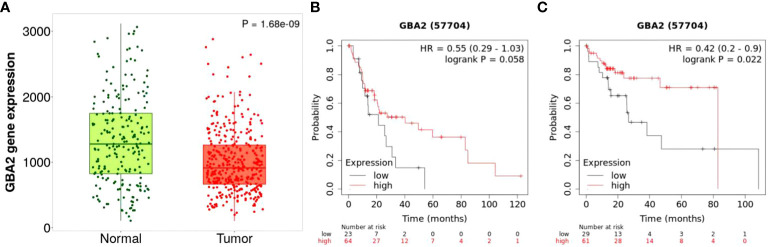
Differential mRNA expression analysis and survival of HCC from the Kaplan-Meier Plotter databases: **(A)** mRNA expression of *GBA2* in HCC tissues and normal liver tissues; **(B)** mRNA expression of *GBA2* relationship with OS in stage 3 + 4 HCC patients; **(C)** mRNA expression of *GBA2* relationship with OS in HCC patients with microvascular invasion.

To clarify the effect of *GBA2* gene expression in the progression and survival of HCC, we first assessed mRNA expression levels of *GBA2* gene in HCC tissues and normal tissues using TNMplot, and analyzed its relationship with OS of HCC patients through Kaplan-Meier Plotter. It was found that the expression level of *GBA2* mRNA in HCC was lower than that in normal liver tissues (**Figure 7A**). Meanwhile, the higher expression levels of *GBA2* seem to be associated with better survival probability in liver cancer patients with microvascular invasion or stage III/IV (**Figures 7B, C**), which is consistent with our additive interaction results.

### Relationship between *GBA2* and immune cell infiltration in HCC

**Figure 8:**
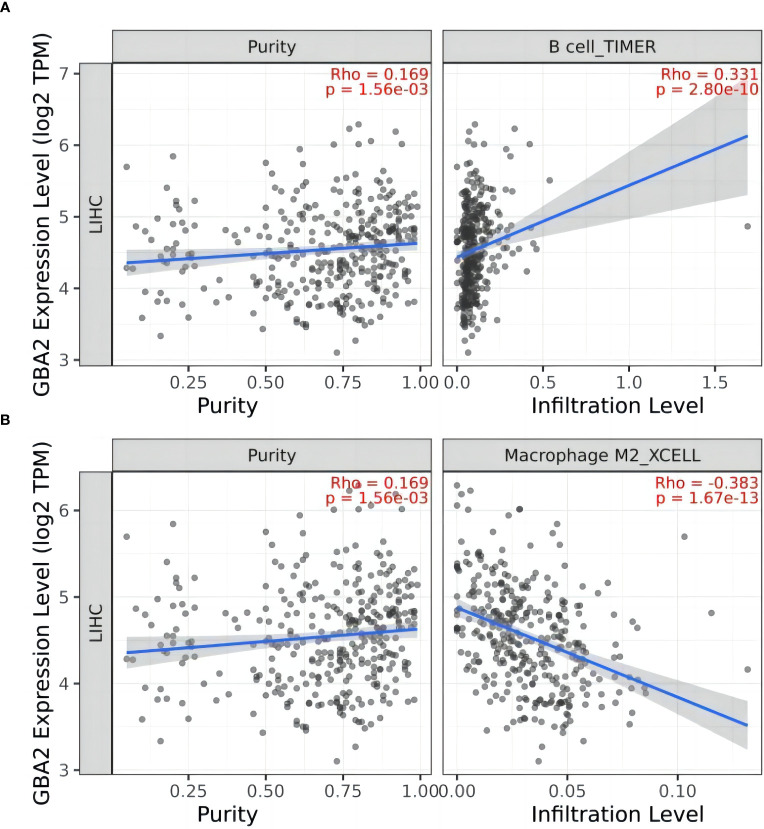
Correlation between *GBA2* mRNA expression and immune cells infiltrating level in HCC tissues: **(A)** B cells; **(B)** Macrophage M2 cells.

To gain further insight into the role of *GBA2* in HCC, we investigated the relationship between *GBA2* and immune cell infiltration in the HCC microenvironment using the TIMER 2.0 database. Our analysis showed that *GBA2* expression was positively correlated with B lymphocyte infiltration levels, and negatively correlated with M2 macrophage infiltration levels (B cell: *R*=0.331, *P*=2.80×10^-10^; Macrophage M2: *R*=-0.383, *P*=1.67×10^-13^) (**Figure 8**). These findings suggest that *GBA2* may modulate immune cell infiltration levels in the tumor microenvironment, which could impact the survival of HCC patients.

## Discussion

In the present study, we evaluated the associations between SNPs in sphingolipid metabolism genes and survival of HBV-HCC patients. Our study identified a novel functional SNP (rs1570247 G>A) located in the 5’UTR of the *GBA2* gene as a protective factor for HCC prognosis. Furthermore, eQTL analysis revealed that the *GBA2* rs1570247 A allele was associated with increased mRNA expression levels, and *GBA2* may play a role in regulating immune cell infiltration in the tumor microenvironment. Overall, our findings suggest that GBA2 rs1570247 G>A in sphingolipid metabolism pathway may have an important impact on survival of HBV-HCC patients, possibly by influencing mRNA expression and affecting the infiltration level of immune cells.

The *GBA2* gene is located on the human chromosome 9 and is mapped in position p13.3 (31). *GBA2* was first isolated and characterized from the human liver, where it was found to hydrolyze endogenous bile acid 3-O-glucosides (32). *GBA2* is a membrane-bound enzyme located in the endoplasmic reticulum(33), and has been found to be present at or near the cell surface(34). It cleaves the β-glucose-sphingosine linkage in glucosylceramide (GlcCer). In addition to its hydrolytic activity on GlcCer, *GBA2* also exhibits transglucosylation activity by transferring glucose to cholesterol. This activity allows *GBA2* to use the glucose moiety released by the cleavage of GlcCer to form glucocholesterol (GlcChol). Conversely, GlcChol can be deglycosylated by *GBA2* to synthesize GlcCer. In this study, we found that *GBA2* rs1570247 A allele was a protective factor for survival in HCC patients and was associated with increased *GBA2* mRNA expression levels. Similarly, *GBA2* mRNA in HCC was lower than that in normal liver tissues, and the higher expression levels of *GBA2* seemed to be associated with better survival probability in liver cancer patients with microvascular invasion or stage III/IV. However, since *GBA2* can cleave glucosylceramide to produce ceramides, ceramides have been shown to act as tumor suppressors in a variety of tumors (35–37). *GBA2* also has been shown to function as a tumor suppressor in melanoma cells and cholangiocarcinoma (38, 39). Meanwhile, based on the role of *GBA2* in inflammatory response (40), we analyzed the relationship between *GBA2* and immune cell infiltration in the tumor microenvironment, and found that *GBA2* was positively correlated with B cells, but negatively correlated with M2 macrophages. B cells arise and mature in the bone marrow and have various functions in immune response (41). Tumor-infiltrating B lymphocytes (TIBs) have been observed in various solid tumors. Studies have shown that TIBs can suppress tumor progression by secreting immunoglobulins, promoting T cell response, and directly killing cancer cells (42). Additionally, TIBs can help induce the infiltration of cytotoxic T lymphocytes (CTL) into tumors by maintaining the structure and function of tertiary lymphoid structures (TLS), which contributes to an effective antitumor response and better patient prognosis (43–45). Macrophages are a crucial component of the tumor microenvironment that facilitate tumor cell migration and invasion, matrix degradation, and angiogenesis. The density of macrophages in the tumor microenvironment has been found to be a prognostic marker of poor outcome for a variety of carcinomas (46–48). M2 macrophages, which are derived from macrophages, have poor antigen presentation ability and produce factors that inhibit the proliferation and activity of T cells. They are typically more suited to promoting angiogenesis, tissue remodeling, and repair (49). Studies have also suggested that M2 macrophages recruited by tumors can facilitate tumor metastasis (50). These findings suggest that *GBA2* may affect the survival of HCC patients by influencing the immune response.

## Conclusion

In conclusion, our study identified the role of genetic variants in *GBA2* (rs1570247 G>A) in HBV-HCC survival. The *GBA2* rs1570247 mutant genotypes were associated with better survival of HBV-HCC, possibly by enhancing *GBA2* transcription and improving the immune response. However, it is important to note that the datasets used to validate our findings only included Chinese populations. Therefore, our results may not be generalizable to other ethnic groups, and further studies are needed to determine the impact on HCC survival in the general population. Additionally, the molecular mechanism of *GBA2* in HCC remains unclear, and further biochemical studies and functional experiments are needed to confirm our findings.

## Data availability statement

The original contributions presented in the study are included in the article/**Supplementary Materials**, further inquiries can be directed to the corresponding author/s.

## Ethics statement

The studies involving humans were approved by Ethics Committee of the Guangxi Medical University Cancer Hospital (LW2023109). The studies were conducted in accordance with the local legislation and institutional requirements. The participants provided their written informed consent to participate in this study.

## Author contributions

Conception and design, HY; Provision of study materials or patients and data collection, BJ, LQ, JT, SZ, JW, JL, YL, YM and JC; Bioinformation analysis, BJ, ZZ, QL and YJ; Follow-up: XL; Data analysis and interpretation, BJ, and MQ; Writing – Original Draft Preparation, BJ, and MQ; Writing – Review and Editing, HY. All authors contributed to the article and approved the submitted version.
