# Supplementary material for: Associations between genetic variants in sphingolipid metabolism pathway genes and hepatitis B virus-related hepatocellular carcinoma survival
Source: Front Oncol. 2024 Jan 8;13:1252158. doi: 10.3389/fonc.2023.1252158 (PMC10801735; doi:10.3389/fonc.2023.1252158)
Supplement: Supplementary file 1 [file Table_1.docx]

**Table S1**. List of 86 selected genes in the sphingolipid metabolism Pathway from MSigDB

| **Dataset** | **Name of pathway ^a^** | **Selected genes** | **Number of genes ^b^** |
| --- | --- | --- | --- |
| REACTOME | SPHINGOLIPID_METABOLISM | *ALDH3B1, ARSD, HEXB, GALC, ARSF, SPHK2, CTSA, PLPP1, GBA2, ALDH3A2, ACER3, SPTLC1, CERS4, ARSA, CERK, SPTLC2, VAPA, STS, GLA, SMPD3, FA2H, ASAH1 ,PRKD2, OSBP, CERT1, ARSB, NEU2, PRKD3, ESYT2, KDSR, ORMDL2, VAPB, SGPP1, ORMDL1, SUMF2, ALDH3B2, CSNK1G2, B4GALNT1, SMPD2, SMPD4, GLTP, CERS5, ESYT1, ARSG, PLPP2, CERS2, DEGS1, SUMF1, UGCG, CERS3, SAMD8, ARSL, ESYT3, NEU3, PLPP3, SGPP2, GLB1L, PPM1L, SGMS2, ARSK, SPTSSA, SGPL1, SMPD1, ACER1, DEGS2, B3GALNT1, GLB1, ORMDL3, CERS6, SPTLC3, UGT8, SPHK1, ACER2, GBA1, ARSJ, ENPP7, SPNS2, ARSI, PRKD1, ASAH2, SPTSSB, GM2A, PSAP, SGMS1, NEU1, ARSH, HEXA, CERS1, CPTP, NEU4* | 90 |
| KEGG | SPHINGOLIPID_METABOLISM | *SPTLC1, NEU3, DEGS2, ACER1, NEU4, SGPP2, SGMS2, KDSR, GALC, SGMS1, GBA1, GLA, GLB1, ENPP7, ACER2, ARSA, ASAH1, NEU1, NEU2, ACER3, SMPD3, SMPD4, ASAH2, SPHK2, CERK, SMPD1, SMPD2, UGCG, UGT8, SGPP1, DEGS1, PLPP1, PLPP2, PLPP3, SPHK1, SGPL1, B4GALT6, GAL3ST1, SPTLC2* | 39 |
| Total | \| After removing 37 duplicate genes and 6 genes on the X chromosome \| \| --- \| | | 86 |

^a^ Genes were selected based on MSigDB

^b^ Duplicate genes, X chromosome and genes that did not match the hg19 gene database were removed

Keyword for MSigDB: Sphingolipid

Organism: Homo sapiens
